# iTRAQ-Based Proteomics of Chronic Renal Failure Rats after FuShengong Decoction Treatment Reveals Haptoglobin and Alpha-1-Antitrypsin as Potential Biomarkers

**DOI:** 10.1155/2017/1480514

**Published:** 2017-04-27

**Authors:** Yu Yang, Junmeng Wei, Xuekuan Huang, Mingjun Wu, Zhenbing Lv, Pan Tong, Rui Chang

**Affiliations:** ^1^College of Traditional Chinese Medicine, Chongqing Medical University, Chongqing 400016, China; ^2^Department of Pathogenic Biology, Chongqing Medical University, Chongqing 400016, China; ^3^College of Life Science and Technology, Chongqing Medical University, Chongqing 400016, China; ^4^The First Clinical Medical College, Chongqing Medical University, Chongqing 400016, China

## Abstract

*Background*. Chronic renal failure (CRF) has become a global health problem and bears a huge economic burden. FuShengong Decoction (FSGD) as traditional Chinese medicine has multiple pharmacological effects.* Objectives*. To understand the underlying molecular mechanism and signaling pathway involved in the FSGD treatment of CRF and screen differentially expressed proteins in rats with CRF treated with FSGD.* Methods*. Thirty-three male Sprague-Dawley rats were randomly divided into control group, CRF group, and FSGD group. Differentially expressed proteins were screened by iTRAQ coupled with nanoLC-MS/MS, and these identified proteins were later analyzed by GO, KEGG, and STRING. Additionally, haptoglobin (HP) and alpha-1-antitrypsin (AAT) were finally verified by ELISA, Western blot, and real time PCR.* Results*. A total of 417 proteins were identified. Nineteen differentially expressed proteins were identified in the FSGD group compared with the model group, of which 3 proteins were upregulated and 16 proteins were downregulated. Cluster analysis indicated that inflammatory response was associated with these proteins and complement and coagulation cascade pathways were predominantly involved. The validation methods further confirmed that the levels of HP and AAT were significantly increased.* Conclusions*. HP and AAT may be the important biomarkers in the pathogenesis of CRF and FSGD therapy.

## 1. Introduction

The incidence of chronic renal failure (CRF) is increasing annually on a global scale [1], thus placing enormous burden on the medical system of many countries [2]. Renal fibrosis characterized a common endpoint of different kidney diseases which resulted in kidney functional impairment ultimately leading to terminal renal failure. Tubulointerstitial fibrosis and glomerulosclerosis were closely associated with diverse action mechanisms such as abnormality of gene and protein expressions [3–6] as well as their downstream low-molecular-weight metabolite dysregulations [7–10]. The relationships of abnormal gene or protein expressions and endogenous metabolite disturbance were demonstrated in diverse chronic renal diseases [11–16]. Current clinical therapies for CRF are scarce and often ineffective [17]. Traditional Chinese medicine is potentially a meaningful alternative therapy for CRF [18–22].

FuShengong Decoction (FSGD) is summarized by Professor of Chinese medicine master Ziguang Guo, who added and deducted some herbs based on the classic formula “jisheng shenqi pills” with 60 years of clinical experience [23]. FSGD is composed of* Radix Astragali*,* Rehmannia glutinosa*,* Dioscorea opposita*,* Fructus Corni*,* Semen Plantaginis*,* Radix Achyranthis Bidentatae*,* Cortex Moutan Radicis*,* Rhizoma Alismatis*,* Poria*,* Rhizoma Atractylodis*,* Cortex Eucommiae*,* Hirudo*, and* Cortex Phellodendri*. Research indicates that* Radix Astragali*, which is the dominate component, plays a role in improving the immunity and renal function [24].* Rehmannia glutinosa* has been shown as an effective constituent that can suppress inflammation and enhance renal function [25].* Dioscorea opposita* has been used to strengthen bone and tonify the kidney [26]. It has been reported that* Rhizoma Alismatis* showed dual effect including promotion and inhibition of diuretic activity on renal function and antihyperlipidemia effect [27–29]. Several studies have demonstrated that* Poria* possessed nephroprotective activities including diuretic activity and treatment of CRF [30–34] and antihyperlipidemia effect [35, 36]. Although the clinical application of FSGD in the treatment of CRF has been verified, the underlying molecular mechanisms of its effect remain unknown.

Isobaric tags for relative and absolute quantitation (iTRAQ) is a technology that can be used to simultaneously measure protein amounts in a multitude of test samples. This method significantly reduces the variability caused by multiple tests, thereby improving the accuracy of qualitative and quantitative protein analyses. iTRAQ has the ability to produce highly accurate and comprehensive information on hundreds to thousands of proteins. In a Pubmed search we found only 173 papers that used iTRAQ labeling to detect serum differential proteins; of these articles none reported on CRF.

In this study, we performed proteomic analysis using iTRAQ technology coupled with nanoscale liquid chromatography tandem mass spectrometry (nanoLC-MS/MS) to unveil molecular mechanism and identify potential biomarkers of FSGD. At the same time, we elucidated potential pathogenesis and the key pathway of these proteins through pathway analysis and protein networks. Furthermore, via the ELISA method, Western blot, and RT-qPCR we verified two dysregulated proteins (HP and AAT) that are of much interest as these two proteins were able to distinguish the CRF levels between model group and FSGD group, and they may act as biomarkers of FSGD.

## 2. Materials and Methods

### 2.1. Substances

FSGD ingredients were selected according to the “Chinese Pharmacopoeia” 2010 Edition. FSGD were soaked for 30 minutes with purified water and boiled three times every 30 minutes for a total of 90 minutes; then the boiling liquid was collected, filtered, concentrated to crude drug with the amount of 1 g/ml, and stored at 4°C for use.

### 2.2. Animals and Sample Collection

A total of 33 male Sprague-Dawley rats (SYXK (Chongqing) 2012-0001), weighting 180 ± 20 g, were fed adaptively for 1 week and then randomly divided into 3 groups: control group, model group, and FSGD group (11 in each group). The control group was fed standard chow, while the other two groups were fed 0.5% adenine (Sigma-Aldrich, St. Louis, MO, USA) chow for 3 weeks to induce chronic renal failure [37, 38]. After the models were successfully made, rats in control and model groups received saline in the amount of 20 ml/kg/d, while those in FSGD group received 16 g/kg/d, administered by gastric irrigation, respectively, for 30 days. All rats were starved for 12 h and anesthetized by 3% pentobarbital (Beijing Propbs Biotechnology, Beijing, China) at a dose of 5 ml/kg at 30 days, and blood samples were later acquired by cardiac puncture. Blood was placed at room temperature for 0.5 hours and was subsequently centrifuged at 4°C, 1300 *g* for 15 min, and the supernatant was obtained and stored at −80°C for further analysis. Kidneys were immediately washed by phosphate buffer saline and stored at −80°C for histological study. The experimental animals were disposed according to the “Guide for the Care and Use of Laboratory Animals” approved by the Committee of Chongqing Medical University.

### 2.3. iTRAQ Labeling

To increase accuracy and reduce variability in measures of protein concentration, the same amount of blood from each group was mixed into one sample [39, 40]. High-abundance proteins such as albumin and IgG were depleted by using the Multiple Affinity Removal System (Agilent, Palo Alto, CA, USA) according to the manufacturer's instructions. Next, proteins were concentrated and desalted [41]. A total of 200 *μ*g proteins were soaked in 6 *μ*l dithiothreitol (Amresco, Solon, OH, USA) and 2 *μ*l indole-3-acetic acid (Amresco, Solon, OH, USA) for 1 h at 37°C and then centrifuged. The deposit was subsequently removed. The samples were then digested with trypsin (AB Sciex, Framingham, MA, USA) with the ratio of protein : trypsin = 50 : 1 at 37°C overnight. The peptides were labeled with iTRAQ reagent (AB Sciex, Framingham, MA, USA) (each reagent was dissolved in 70 *μ*l of ethanol) and incubated at room temperature for 2 h. The samples were labeled as follows: the control group, 113; the model group, 114; the FSGD group, 115; then they were mixed and dried by vacuum centrifugation. To avoid the labeling bias, two independent biological replicates were performed.

Strong cation exchange (SCX) chromatography was performed to separate protein with the LC-20AB HPLC Pump system (Shimadzu, Kyoto, Honshu, Japan) with Gemini-NX C18 column (Phenomenex, Torrance, CA, US) (4.6 × 250 mm, 5 *μ*m 110A). The peptide mixture was eluted with a liner gradient of buffer A (AB Sciex, Framingham, MA, USA) and 5% buffer B (AB Sciex, Framingham, MA, USA) for 30 min, 15%–90% buffer B for 25 min, and 5% buffer B for 10 min at a flow of 0.8 ml/min. A total of 50 components were collected and vacuum-dried.

### 2.4. Mass Spectrometry (MS) Analyses

The fractions were centrifuged at 12,000 *g* for 8 min and supernatant was collected. The samples were analyzed with nanoHPLC-MS/MS (Thermo Scientific, Waltham, MA, USA). Specific parameters were as follows: ion spray voltage, 2.3 kv; Curtain gas, 35 psi; survey scan, 300–1800 *m*/*z* for MS scans; dynamic exclusion duration of 25 s; survey scan, 100–1500 *m*/*z* for MS/MS scans.

Peptide and protein identification were performed using the ProteinPilot™ software (version 4.2; Applied Biosystems, USA) and searching an automated database against the rat database (IPI_rat_v3.87) with the Mascot search engine (version 2.3.02; Matrix Science, London, UK). To screen the differential proteins, the threshold was applied as follows: the unused ProtScore > 1.3 and at least one peptide with a 95% confidence level [42], ratios with fold change > 1.2 (or <0.83), and *P* values < 0.05 were considered to be significant.

### 2.5. Bioinformatics Analysis

Gene ontology (GO) analysis and the Kyoto encyclopedia of genes and genomes (KEGG) database were used to enrich and cluster the differential proteins. Each protein was represented by its cellular components, molecular function, and biological process by the GO database; meanwhile the pathway analysis was performed using the KEGG database. Functional networks were determined by STRING protein-protein interaction networks. All of the above analyses were conducted with Omicsbean software (Geneforhealth, Shanghai, China).

### 2.6. ELISA Methods

Rat HPT ELISA kit (Abcam, Cambridge, MA, USA, SwissProt: P06866) and rat *α*-1-AT ELISA kit (Abcam, Cambridge, MA, USA, SwissProt: P17475) were used to detect protein levels in the serum. The protein concentration analysis of each group was performed according to the manufacturer's protocols. Concentrations in each group were compared with Student's *t*-tests after logarithmic transformation.

### 2.7. Real Time Quantitative PCR

The renal samples stored at −80°C were uniformized in TRIzol (Tiangen Biotech, Beijing, China) and the RNA extraction was performed according to the manufacturer's directions. Then the RNA was transcribed into cDNA (Toyobo, Shanghai, China) according to the manufacturer's protocols. The PCR reaction was submitted to CFX96 Touch Real Time PCR (Bio Rad, Hercules, CA, USA) with the following primers: rat* HP*: sense, 5′-TGTGCCGTAGCTGAGTATGGTGTG-3′, antisense, 5′-GAATTGCCCTGCCCCACTGT-3′, rat* Serpina1*: sense, 5′-CCCTTGGCGACCCTCCTCTT-3′, antisense, 5′-CCCCACCGAAGAACCAGGATATA-3′, and *β*-actin: sense, 5′-ACCCCGTGCTGCTGACCGAG-3′, antisense, 5′-TCCCGGCCAGCCAGGTCCA-3′ according to the manufacturer's instructions. Afterwards, the expression of genes was calculated from standard curve with the expression of *β*-actin gene as reference.

### 2.8. Western Blot Analysis

The proteins were separated from frozen renal tissues and the protein concentration was measured by bicinchoninic acid (BCA) assay kit (Thermo Fisher Scientific, Rockford, IL, USA). The protein samples were resolved and transferred onto polyvinylidene fluoride (PVDF) membranes. After blocking with 5% nonfat milk at room temperature for 2 h, the membranes were performed using specific primary antibody as follows: haptoglobin and alpha-1-antitrypsin (Abcam, Cambridge, MA, USA). The blots were incubated with horseradish peroxidase-conjugated secondary antibodies (Abcam, Cambridge, MA, USA) and then exposed with an ECL kit (GE Healthcare, Chicago, IL, USA).

### 2.9. Statistical Analysis

Statistical analyses were performed with GraphPad Prism software version 5.01 (GraphPad Software, Inc., San Diego, CA, USA). Variables in each group were tested to determine if they were normally distributed. Multiple comparisons of sample means were used for analysis of variance. The SNK method was used for pairwise comparison. *P* < 0.05 was considered to be significant.

## 3. Results

### 3.1. Comparative Analysis of Serum Proteomic Changes in Each Group

The overall proteins were compared among the three groups. In total, 417 proteins were confirmed with 5% local false discovery rate (FDR) and > 95% confidence score. Nineteen proteins with differential expression were found using stringent criteria. Among these proteins, twelve were found to be upregulated in the models compared with the controls and these same proteins were found to be downregulated in the FSGD group compared with the models. Additionally two downregulated proteins were then shown to be upregulated in the FSGD group, and five proteins exhibited no significant differences between groups. In addition, the levels of five proteins showed no difference between the controls and FSGD group. It is worth mentioning that the fold changes of HP were striking after FSGD treatment (Table 1).

### 3.2. GO Analysis, KEGG Pathway, and STRING

**Figure 1:**
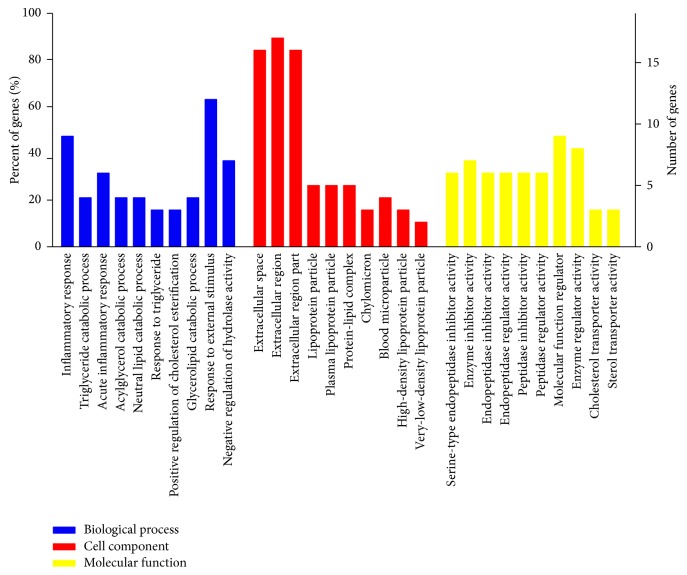
GO analysis of differentially expressed proteins.

Differentially expressed proteins of the FSGD and model groups were catalogued based on GO enrichment analysis. It was revealed that most of the proteins were involved in the response to external stimulus (12, 63.16%), inflammatory response (9, 47.37%), and negative regulation of hydrolase activity (7, 36.84%). In addition, the subcellular proteins were distributed in the extracellular region (17, 89.47%), extracellular region part (16, 84.21%), and extracellular space (16, 84.21%) and associated with molecular function regulators (9, 47.37%), enzyme regulator activity (8, 42.11%), and enzyme inhibitor activity (7, 36.84%) (Figure 1).

**Figure 2:**
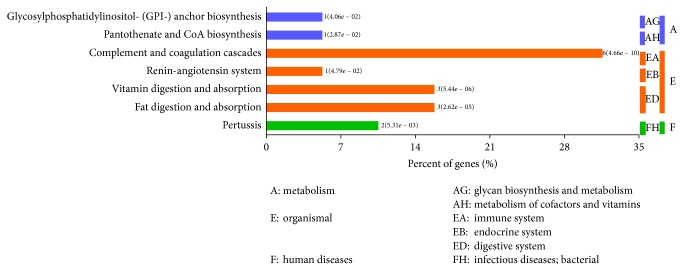
KEGG pathway mapping of differentially expressed proteins.

The KEGG pathway mapping indicated that complement and coagulation cascades (6 proteins) were the predominant pathways. Vitamin digestion and absorption (3 proteins) and fat digestion and absorption (3 proteins) were also verified; these proteins are associated with the immune, endocrine, and digestive systems (Figure 2).

**Figure 3:**
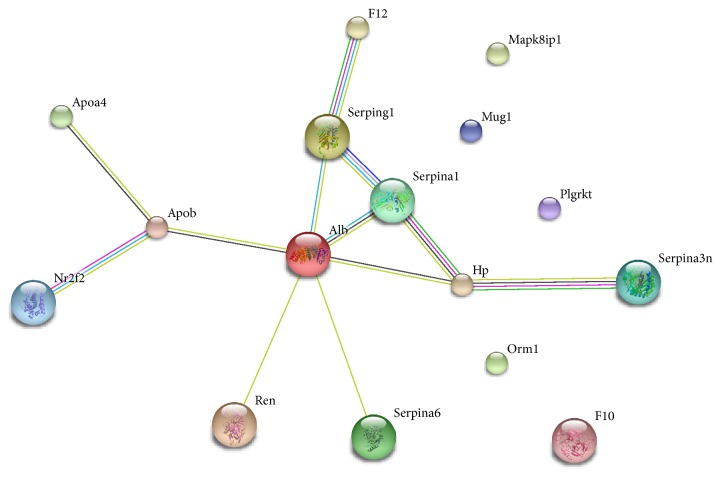
The network of differentially expressed proteins obtained by STRING analysis.

The interactions among the differentially proteins were analyzed by the STRING network (Figure 3). STRING analysis showed that Alb and Serpina1 played a central role in the network.

### 3.3. The Validation of ELISA, Western Blot, and RT-qPCR

**Figure 4:**
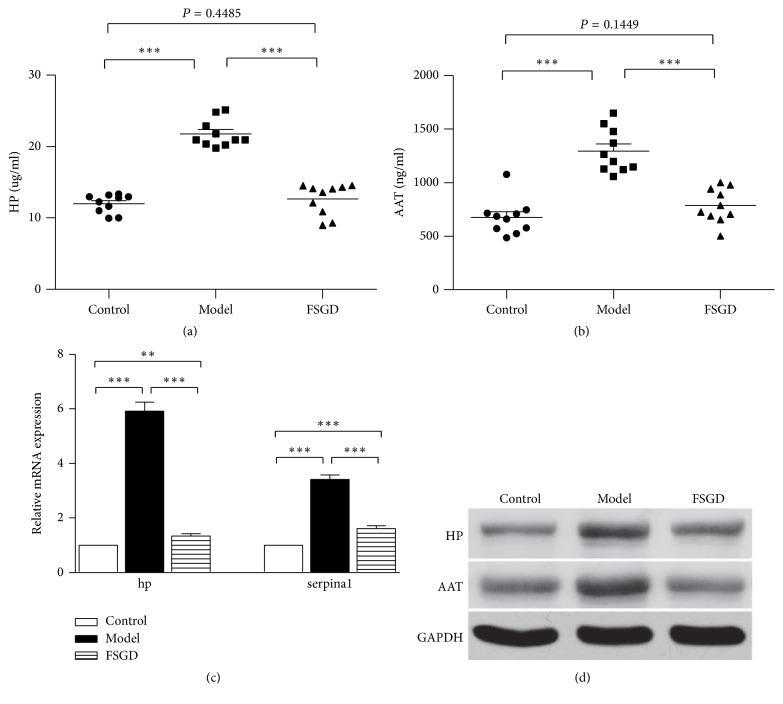
The expressions of HP and AAT among the control, model, and FSGD groups obtained by the ELISA, Western blot, and RT-qPCR methods. ((a) and (b)) The result of ELISA method. (c) The expression of each gene by RT-qPCR. (d) Western blot analysis for HP and AAT. (e) Densitometric analysis for HP and AAT. A *P* value less than 0.05 indicates statistical significance using the *t*-test. ^*∗∗∗*^*P* < 0.001; ^*∗∗*^*P* < 0.01. The bars represent the means ± standard deviations of ten rats.

Based on the central role in the STRING network and the fold changes, we selected HP and AAT for further analysis. The results revealed that both HP and AAT levels were significantly increased (*P* < 0.001, *P* < 0.001, resp.) in the control group compared with the model group. HP and AAT levels were significantly decreased (*P* < 0.001, *P* < 0.001, resp.) in the model group compared to the FSGD group. In addition, there were no significant differences in HP and AAT levels between the model group and the FSGD group (*P* = 0.4485, *P* = 0.1449, resp.) (Figure 4).

## 4. Discussion

We examined the therapeutic effect of FSGD in the adenine-induced CRF rats. In traditional Chinese medicine, compounds of Chinese herbs have long-standing and widespread clinical applications. Multiple components of different herbs can concurrently attack multiple targets involved in the pathogenesis of the diseases. Thus, compounds are more important than a single herb [19, 43]. Previous studies have verified that FSGD is effective in the treatment of CRF. Specifically, levels of serum creatinine (SCr) and blood urea nitrogen (BUN) decreased significantly; renal function and nephridial tissues were improved after treatment of FSGD [44, 45]. We found that possible mechanisms may be the inhibition of Sonic Hedgehog (SHH) signaling pathway and/or reduced levels of *α*-SMA in nephridial tissue by detecting renal tissues. To characterize the effect of FSGD, we conducted the present experiment.

We initially examined the mechanisms of the adenine-induced CRF. CRF is similar to chronic renal insufficiency and chronic kidney disease. Several previous studies have showed that diabetic nephropathy [46, 47], hypertensive nephropathy [48, 49], and lupus nephritis [50] are the primary causes of CRF. Inagi [51] found that the accumulation of advanced glycation end product (AGE) produces glycative stress closely associated with kidney disease. Various signaling pathways are involved in process of chronic kidney disease, such as Wnt/*β*-catenin, TGF-*β*/Smads, JNK/STAT3, and MAPKs [52]. The common pathway of these renal diseases is tubulointerstitial fibrosis, which is characterized by the superfluous deposition of extracellular matrix, infiltration of lymphocytes, dendritic cells, macrophages [53], and fibroblast proliferation/differentiation.

By function and pathway analysis, our study demonstrated that complement and coagulation cascade pathways and inflammatory response have a striking response on protein structure. The complement system is composed of over 30 serum proteins and cell membrane proteins [54], appears congenitally and/or is an acquired immune effector, and is one of the most powerful barriers against invading pathogens. The deposition of immune complexes mediated inflammatory response causing tissue injury [55]. In addition, coagulation factors are activated after the interaction of platelets and endotheliocyte, thus enhancing coagulation and inflammation [56]. Keir and Langman [54] also indicated that complement factors are related to kidney function. Further, activation of the complement system may aggravate kidney damage. Hence, we assume that the development of CRF accompanied activation of the complement system and inflammatory infiltration.

Haptoglobin (HP), as an acute phase protein, exists as two major alleles: HP1 and HP2. The main efficacy of HP1 is antioxidant and anti-inflammatory, while HP2 plays an important role in antagonism. The main function of HP is to combine with free hemoglobin (Hb) and bind to monocytic cells and lymphocytes, thereby avoiding the loss of the Hb and heme iron from the kidney and damage to the kidney [57, 58]. Several studies have demonstrated that higher levels of HP are correlated with liver fibrosis [59], various cancers [60–62], and cardiovascular disease [63]. HP is mainly produced in the liver but also expressed in kidney. The common mechanism of these diseases indicates that HP is associated with inflammation and the immune response [57]. In addition, several previous studies have showed that HP can predict various kidney diseases [64–66]. Specifically, previous reports show that HP2 is more closely related to kidney disease. Elevated concentrations of HP2 in the serum have been shown to increase deterioration of renal function [67]. HP response to injury or improved oxidative stress could be expressed in the renal tubules. This phenomenon may contribute to development of haptoglobin-hemoglobin complex that cannot be filtered from the glomerulus and, along with iron, accumulates in renal proximal tubule. This is a possible mechanism of CRF. Our proteomic result further demonstrates that HP is critical in the progress of CRF.

Alpha-1-antitrypsin (AAT), as the serine proteinase inhibitor, can prevent pathological damage of tissue, inhibit infection and inflammation, and organize and maintain the internal environment of body [58]. AAT has multiple activities, such as cytoprotective, immunomodulatory activity and downregulation of neutrophil elastase during the inflammatory processes. Accumulating studies have showed that AAT may lead to lung pathologies [68], type 1 diabetes [69], arthritis [70], and lupus [71]. Kwak et al. [72] found that the expression of AAT is elevated in renal biopsies, while other investigators detected that it is overexpressed in the urine of some renal diseases [73–75]. This phenomenon was consistent with our result in which AAT was upregulated. Upregulation of AAT could lead to inhibition of elastase, which can contribute to regulating inflammation and the accumulation of mesangial matrix, as well as maintaining the elasticity of blood vessels and glomerular integrity. AAT is located in the cytoplasm of podocytes and it is possibly related to epithelial dysfunction and podocyte stress and results in renal fibrosis.

In conclusion, we succeeded in finding differentially expressed proteins in the adenine-induced CRF. According to the function and pathway analyses, it was demonstrated that these proteins are involved in multiple pathways and biological processes, but mainly in the inflammatory response. The results are consistent with the multitarget way of traditional Chinese medicine. Interestingly, HP and AAT exhibited significantly changes and located in key positions. This finding was verified by ELISA and the results were consistent with serum proteomics. We presumed that HP and AAT could be applicable as markers in the progression of CRF and may be the candidate biomarkers of FSGD. Further research is needed to explore the role of these protein functions in pathogenesis.

## Figures and Tables

**Table 1:** Differentially expressed proteins among control, model, and FSGD groups obtained by iTRAQ-nanoHPLC-MS/MS. 113: control; 114: model; 115: FSGD; —: ratio between 0.83 and 1.20.

Protein ID	Protein name	115:114	114:113	115:113
sp∣P06866	Haptoglobin	0.10	12.62	1.74
sp∣P04639	Apolipoprotein A-I	0.17	4.90	—
sp∣Q6P734	Plasma protease C1 inhibitor	0.19	1.80	0.32
sp∣P02651	Apolipoprotein A-IV	0.23	2.28	0.52
sp∣P02764	Alpha-1-acid glycoprotein	0.25	1.87	0.45
sp∣P09006	Serine protease inhibitor A3N	0.28	1.71	0.48
tr∣D4A183	Protein Vnn3	0.35	—	0.31
sp∣Q03626	Murinoglobulin-1	0.35	—	0.40
sp∣Q63207	Coagulation factor X	0.36	—	0.37
sp∣D3ZTE0	Coagulation factor XII	0.43	1.84	0.79
tr∣Q68FY4	Group specific component	0.45	2.22	—
sp∣P17475	Alpha-1-antiproteinase	0.46	2.74	1.25
tr∣A9CME3	Complement component 4 binding protein, alpha	0.49	2.20	—
sp∣P01015	Angiotensinogen	0.49	2.72	1.46
tr∣G3V8B1	Glycosylphosphatidylinositol specific phospholipase D1, isoform CRA_a	0.58	—	0.67
tr∣F1M6Z1	Apolipoprotein B-100	0.73	4.60	3.30
sp∣P02770	Serum albumin	1.28	0.79	—
sp∣P31211	Corticosteroid-binding globulin	2.24	1.79	3.48
sp∣Q01177	Plasminogen	2.90	0.35	—
